# Topological quantum phase transition from mirror to time reversal symmetry protected topological insulator

**DOI:** 10.1038/s41467-017-01204-0

**Published:** 2017-10-17

**Authors:** Partha S. Mandal, Gunther Springholz, Valentine V. Volobuev, Ondrej Caha, Andrei Varykhalov, Evangelos Golias, Günther Bauer, Oliver Rader, Jaime Sánchez-Barriga

**Affiliations:** 1 Helmholtz-Zentrum Berlin für Materialien und Energie, Albert-Einstein Strasse 15, 12489 Berlin, Germany; 2 Institut für Physik und Astronomie, Universität Potsdam, Karl-Liebknecht Street 24/25, 14476 Potsdam, Germany; 3 Institute for Semiconductor and Solid State Physics, Johannes Kepler Universität, Altenberger Strasse 69, 4040 Linz, Austria; 4 National Technical University “Kharkiv Polytechnic Institute”, Frunze Street 21, 61002 Kharkiv, Ukraine; 5 Department of Condensed Matter Physics, Masaryk University, Kotlářská 267/2, 61137 Brno, Czech Republic

## Abstract

Topological insulators constitute a new phase of matter protected by symmetries. Time-reversal symmetry protects strong topological insulators of the Z_2_ class, which possess an odd number of metallic surface states with dispersion of a Dirac cone. Topological crystalline insulators are merely protected by individual crystal symmetries and exist for an even number of Dirac cones. Here, we demonstrate that Bi-doping of Pb_1−*x*_Sn_*x*_Se (111) epilayers induces a quantum phase transition from a topological crystalline insulator to a Z_2_ topological insulator. This occurs because Bi-doping lifts the fourfold valley degeneracy and induces a gap at $\bar \Gamma$, while the three Dirac cones at the ${\bar{\rm M}}$ points of the surface Brillouin zone remain intact. We interpret this new phase transition as caused by a lattice distortion. Our findings extend the topological phase diagram enormously and make strong topological insulators switchable by distortions or electric fields.

## Abstract (web summary)

Transitions between topological phases of matter protected by different symmetries remain rare. Here, Mandal et al. report a quantum phase transition from a topological crystalline insulator to a Z_2_ topological insulator by doping Bi into Pb_1-*x*_Sn_*x*_Se (111) thin films.

## Introduction

Topological insulators are bulk insulators with a metallic surface state^1^. Provided that the system stays in the same symmetry class of the Hamiltonian^2^, it is fundamentally impossible to follow a path from this topologically distinct phase of matter to a trivial phase without closing the insulating bulk band gap. For a strong topological insulator, metallic surface states are necessarily present at the boundary to a trivial insulator as, for example, air or vacuum. These topological surface states are protected by time-reversal symmetry and their energy vs. momentum dispersion mimics quasirelativistic, massless particles, with the shape of a Dirac cone and a peculiar helical spin texture^1–4^. The topological classification is given by the so-called Z_2_ invariant *ν*_0_, which for odd number of Dirac cones is *ν*_0_ = 1, giving rise to strong (Z_2_) topological insulators, but for even number of cones is zero, characterizing weak topological or trivial insulators^5, 6^.

It is possible to transform a strong (Z_2_) topological insulator to a trivial insulator by alloying as has been shown for Bi_2_Se_3_:In^7, 8^ and BiTl(S_1−*x*_Se_*x*_)_2_^9^. The fundamental principle of bulk-boundary correspondence dictates again that this topological phase transition proceeds through a transition point where the bulk band gap closes. In this picture, it is supposed that the crystal symmetry is maintained through the phase transition. However, the crystal symmetry itself can protect topologically distinct phases as well, termed topological crystalline insulators (TCIs)^5, 10–14^. For TCIs, the decisive role of the crystal symmetry renders the topological protection dependent on the specific crystal face^10^. The topological invariants allow for an even number of Dirac cones, which are, however, not robust against disorder^5^. Pb_1−*x*_Sn_*x*_Se and Pb_1−*x*_Sn_*x*_Te represent such mirror-symmetry protected TCIs with fourfold valley degeneracy^5, 11^ in which the trivial-to-TCI phase transition is reached for sufficiently large Sn contents^10, 15^. Upon cooling, the lattice contracts and the enhanced orbital overlap leads to an inverted (i.e., negative) bulk band gap, which, via bulk-boundary correspondence, gives rise to Dirac cone surface states. This has impressively been shown by temperature-dependent angle-resolved photoemission (ARPES)^15^.

On the other hand, there are crystals that are not necessarily topological, but change their symmetry and their electronic properties with temperature. Such phase-change materials have been studied intensively for non-volatile data storage because their properties can be altered dramatically at the structural phase transition^16^. GeTe is such a prototypical material^16, 17^ that transforms upon cooling from the cubic rock salt to a rhombohedral structure characterized by a large relative sublattice displacement. This gives rise to pronounced ferroelectricity of GeTe^18^ and has recently been found to allow for an electrical switching of electronic properties^19^. Symmetry changes are potentially very interesting also for topological insulators. In fact, it has been shown for the TCI Pb_1−*x*_Sn_*x*_Se that by breaking of mirror symmetries two out of the four Dirac cones at its (100) surface can be gapped^11, 20–22^. In that case, the topological phase remains, however, unchanged by the symmetry breaking and the Z_2_ invariant stays even. It is crucial for the present work to note that in principle the topological phase does not need to be preserved by a distortion. Indeed, topological phase transitions have recently been predicted for two-dimensional TlSe^23^ and three-dimensional SnTe by distortions^24^ and by finite-size effects^25^.

Here, we investigate the band topology of the (111) surface of Pb_1−*x*_Sn_*x*_Se by cooling through the complete trivial to topological phase transition. We demonstrate a new type of phase transition from crystal-symmetry-protected to time-reversal symmetry-protected topology controlled by Bi incorporation. We show that when Bi is introduced in the bulk making the system *n*-type, a gap is opened up at the one Dirac cone at the center of the surface Brillouin zone at $\bar \Gamma$_,_ while the three Dirac cones located at the ${\bar{\rm M}}$ points at the zone boundaries behave as in pure Pb_1−*x*_Sn_*x*_Se, that is, are gapless at low temperature. Our findings provide the first experimental evidence for a topological phase transition from a TCI with an even number of Dirac cones to a Z_2_ time-reversal symmetry-protected strong topological insulator where the number is odd (three). Following the recent prediction^24^, the origin of the novel topological phase transition is interpreted as due to a sublattice shift and rhombohedral distortion along the [111] direction, which lifts the bulk band inversion only at the *Z*-point (*L*-point in the undistorted phase) projected onto $\bar \Gamma$. At the same time, we do not find any evidence for a bulk band gap closing across the phase transition, most likely, because the rhombohedral distortion does not leave the system in the same symmetry class where the TCI is defined.

## Results

### Effect of temperature and Sn concentration

**Fig. 1:**
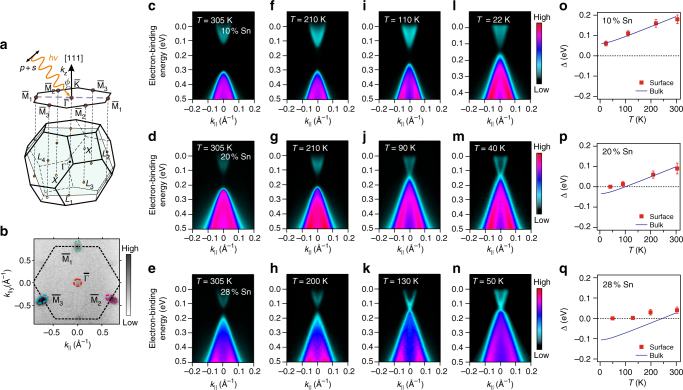
Trivial to TCI phase transition induced by cooling. The phase transition into a topological crystalline insulator (TCI) is monitored for undoped Pb_1−*x*_Sn_*x*_Se (111) films by ARPES around the $\bar \Gamma$ point. **a** For (111) films, the four bulk *L*-points of the bulk Brillouin zone project onto the surface $\bar \Gamma$-point for the longitudinal *L*-valley along [111], whereas the three oblique valleys project on the ${\bar{\rm M}}$ points. **b** At these momenta, Dirac points appear in ARPES. **c**–**q** ARPES data measured at 18 eV photon energy as a function of temperature and Sn content. The *x*_Sn_ = 10% sample (**c**, **f**, **i**, and **l**) remains trivial down to low temperature as seen from the persistence of a band gap. For *x*_Sn_ = 20% (**d**, **g**, **j**, and **m**) and 28% (**e**, **h**, **k**, and **n**), gapless Dirac cones develop at low temperature due to the inversion of the bulk band gap. Panels **o**–**q** show the comparison of the measured surface (Dirac cone) band gap (red squares) with the bulk band gap (blue line) from optical data^27^, evidencing that the surface gap closes when the bulk band gap changes sign. The error bars in the measured surface band gap correspond to the uncertainty in determining the energy position of the band dispersions at the $\bar \Gamma$-point (see Supplementary Note 6 for details). The ARPES dispersions were acquired using linearly polarized *p* + *s* photons incident on the sample under an angle *ϕ * = 45° as shown in **a** (see also Supplementary Note 5)

We have grown both undoped and Bi-doped epitaxial (111) Pb_1−*x*_Sn_*x*_Se films of high quality by molecular beam epitaxy (see Supplementary Fig. 1–6 and Supplementary Notes 1–4 for details). The samples were capped in-situ by a thin Se layer to protect the surface during transport to the ARPES setup, where the cap was desorbed by annealing (see also “Methods” section and Supplementary Note 5). ARPES measurements were performed using linearly polarized light incident on the sample under the geometry shown in Fig. 1a, which also depicts the bulk and (111) surface Brillouin zones of rock salt Pb_1−*x*_Sn_*x*_Se. In contrast to the natural (100) cleavage plane of bulk crystals previously studied^12, 13, 15, 26^, for the (111) orientation, the four bulk *L*-points project on the following four time-reversal invariant surface momenta: $\bar \Gamma$ and three equivalent ${\bar{\rm M}}$ points^27^. This is seen in the ARPES data shown in Fig. 1b, where the intensity from the Dirac cones at the three ${\bar{\rm M}}$ points is enhanced by a photoemission final-state effect. Due to the sensitive dependence of the bulk band inversion on the lattice constant, the trivial to topological phase transition can be monitored during cooling by tracing the evolution of the bulk band gap^28^ or by observation of the appearance of the Dirac cones in ARPES^15, 26^. Indeed, as seen in Fig. 1c–q, for our (111) films with low Sn concentrations and without Bi doping (*x*_Sn_ = 10%, Fig. 1c, f, i, and l), the Dirac cone at the $\bar \Gamma$ point does not form down to 22 K, while for *x*_Sn_ = 20% (Fig. 1d, g, j, and m) and *x*_Sn_ = 28% (Fig. 1e, h, k, and n), the gapless Dirac cone appears at around 90 and 130 K, respectively.

### Impact of Bi incorporation in the bulk

**Fig. 2:**
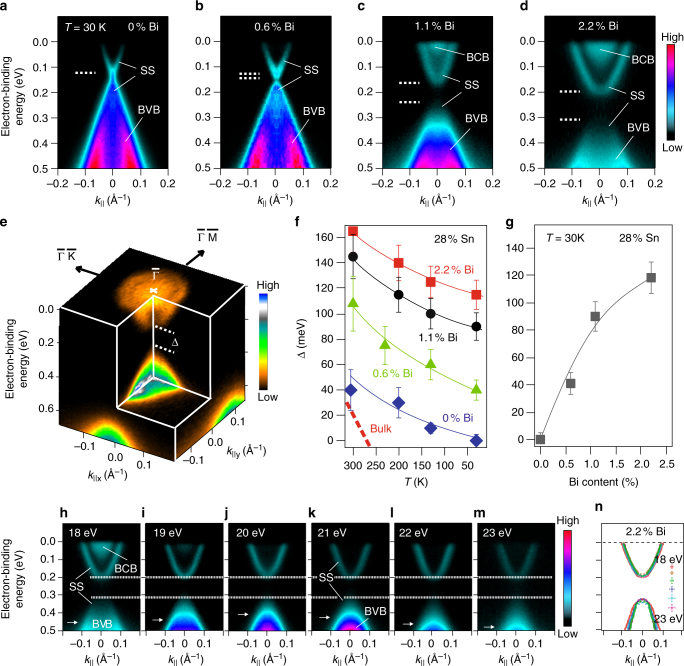
Doping effect and gap opening at $\bar \Gamma$ induced by Bi. Incorporation of Bi in Pb_0.72_Sn_0.28_Se (111) films leads to *n*-type doping and a gap opening at the $\bar \Gamma$ point as revealed by ARPES. **a**–**d** ARPES data measured at a temperature of 30 K and 18 eV photon energy. **e** Corresponding full ARPES map around the $\bar \Gamma$ point for *n*_Bi_ = 2.2%. **f**, **g** Dependence of the surface band gap Δ on temperature and *n*_Bi_. The error bars correspond to the uncertainty in determining the energy position of the band dispersions at the $\bar \Gamma$-point (Supplementary Note 6). **h**–**m** Photon energy dependence for *n*_Bi_ = 2.2%, indicating that the gapped surface state (SS) is two-dimensional. At high binding energies, the dispersion of the bulk-valence band (BVB) with photon energy (marked with horizontal white arrows) can be distinguished from the lower half of the SS. The dispersion of the bulk-conduction band (BCB) can be observed between 18 and 20 eV, while the size of the surface gap remains qualitatively unchanged as indicated by horizontal dashed lines. **n** Energy-momentum dispersions of the upper and lower part of the SS as extracted from fits to results shown in **h**–**m** (see Supplementary Fig. 8 and Supplementary Note 7 for details). The error bars given in the legend represent the maximum uncertainty in determining the corresponding band dispersions

Figure 2 shows the effect of bulk Bi doping on the $\bar \Gamma$ Dirac cone of Pb_0.72_Sn_0.28_Se. When substitutionally incorporated at cation (Pb, Sn—group IV) lattice sites, the group V element Bi acts as electron donor due to its excess valence electron^29^. Increasing the Bi concentration *n*_Bi_ thus leads to a strong upward shift of the Fermi level by 200 meV for *n*_Bi_ = 2.2% (Fig. 2a–e). Remarkably, while for undoped Pb_0.72_Sn_0.28_Se, an intact Dirac cone is seen at 30 K, a surface gap as large as ~100 meV opens up at the $\bar \Gamma$ Dirac cone upon Bi doping and increases strongly with increasing Bi content (see Fig. 2f, g, Supplementary Fig. 7, and Supplementary Note 6). This leads to the general conclusion that for Bi concentrations >~0.6%, the gap at $\bar \Gamma$does not close. Figure 2h–n demonstrates that within the experimental error bars, this gapped surface state does not disperse with the photon energy, i.e., momentum perpendicular to the surface plane, pinpointing its two-dimensional nature (see also Supplementary Fig. 8 and discussion in Supplementary Note 7). This observation is important to rule out that the probed state corresponds to the gapped bulk states.

**Fig. 3:**
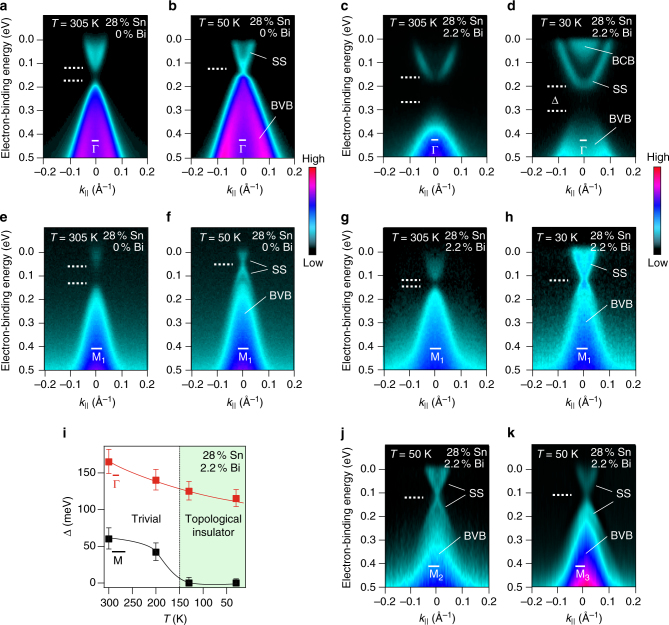
TCI to Z_2_ topological phase transition induced by Bi. **a**–**k** ARPES data of Pb_1−*x*_Sn_*x*_Se (111) films recorded using 18 eV photon energy at $\bar \Gamma$ and ${\bar{\rm M}}$ without (**a**, **b**, **e**, and **f**) and with high Bi doping (**c**, **d**, **g**, **h**, **j**, and **k**). Without Bi doping (**b**, **f**), the Dirac cones simultaneously close at $\bar \Gamma$ and ${\bar{\rm M}}$ <150 K, whereas for high Bi concentration, the Dirac cone is gapped at $\bar \Gamma$ (**d**) and intact at all three ${\bar{\rm M}}$ points at low temperatures (**h**, **j**, and **k**). **i** Temperature dependence of the gap Δ at $\bar \Gamma$ (red) and ${\bar{\rm M}}$ (black). Solid lines are a guide to the eye, and the error bars correspond to the uncertainty in determining the energy position of the band dispersions at $\bar \Gamma$ and ${\bar{\rm M}}$, respectively

The effect of Bi on the Dirac cones at the $\bar \Gamma$ and ${\bar{\rm M}}$ points was evaluated in dependence of the Bi and Sn contents as well as of temperature. On the topologically trivial side (*x*_Sn_ < 16%), for Bi doping *n*_Bi_ < 0.6%, no difference in the gaps Δ at $\bar \Gamma$ and ${\bar{\rm M}}$ is found, which remain open at all temperatures (Supplementary Figs. 9, 10, and Supplementary Note 8). On the topologically non-trivial side (*x*_Sn_ > 16%), the Dirac cones at $\bar \Gamma$ (Fig. 3a–d) and ${\bar{\rm M}}$ (Fig. 3e–h) close synchronously as a function of temperature for low Bi concentrations. This is shown by Fig. 3a, b, e, and f, where corresponding ARPES dispersions at 305 and 50 K are presented. In contrast, for high Bi concentrations (Fig. 3c, d, g, and h), the fourfold valley degeneracy is completely lifted such that the gap is closed to zero at all three ${\bar{\rm M}}$ points (Fig. 3h–k), but opens as wide as 100 meV at the $\bar \Gamma$ point at 30 K (Fig. 3d). Thus, we conclude that of the even numbered Dirac cones per surface Brillouin zone, characteristic of a TCI, only three remain, qualifying Pb_1−*x*_Sn_*x*_Se:Bi as a strong Z_2_ topological insulator already for moderate Bi doping. This is the central result of the present work. While a TCI is protected by mirror symmetry, the odd numbered Dirac cones of strong topological insulators are protected by time-reversal symmetry and robust against disorder. Figure 3i shows that the new Z_2_ phase, which has never been observed before for this material class, exists in a wide temperature range from ~150 K down to the lowest temperature probed in our experiments.

## Discussion

**Fig. 4:**
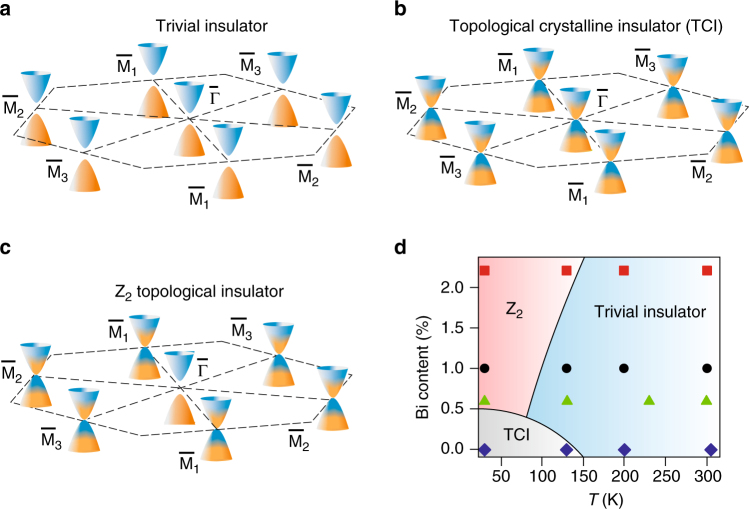
Topological phase transition as a function of Bi doping and temperature. Schematic illustration of the transition from (**a**) trivial to (**b**) topological crystalline insulator (TCI) and eventually to (**c**) strong Z_2_ topological insulator. **d** Topological phase diagram derived from our ARPES data for *x*_Sn_ = 28%. The phase diagram contains a trivial phase at high temperature (positive gap Δ at all four *L*-points) with massive gapped cones at $\bar \Gamma$ and ${\bar{\rm M}}$; a TCI phase at low temperature with even Z_2_ invariant (all bulk *L*-points have negative, i.e., inverted gaps) and closed Dirac cones at $\bar \Gamma$ and all ${\bar{\rm M}}$ points; a Z_2_ topological insulator phase with odd Z_2_ invariant due to a distortion along [111], giving three closed Dirac cones at the ${\bar {\rm M}}$ points and an open gap at the $\bar \Gamma$ point

Due to the bulk-boundary correspondence, the formation of the Dirac cones at the surface indicates a bulk band inversion at the parent momenta in the Brillouin zone as indicated by Fig. 1a. The band structures and shape of the Dirac cones corresponding to the different topological phases are illustrated schematically in Fig. 4a–c, where the open and closed Dirac cones are colored such as to indicate the changes of the predominant charge density at the anion (orange) and cation sites (blue) occurring during the band inversion (see Supplementary Fig. 11 and Supplementary Note 9). The topological phase transition so far reported for Pb_1−*x*_Sn_*x*_Se^15, 26^ occurs between trivial (Fig. 4a) and TCI phase (Fig. 4b) where all bulk band inversions behave equally. For the new topological phase transition into the Z_2_ topological insulator phase (Fig. 4c), the bulk band inversion along the momenta normal to the surface does not occur, triggered by the Bi doping. This means the discovery of two new topological phase transitions in Pb_1−*x*_Sn_*x*_Se: (i) from trivial to Z_2_ topological by cooling and (ii) from topological crystalline to Z_2_ topological by adding Bi. The resulting topological phase diagram (Fig. 4d) derived from our data shows the interdependence of these two pathways.

The observed behavior resembles the predictions by Plekhanov et al.^24^ for SnTe. SnTe is known for its ferroelectric phase transition in which the anion and cation sublattices are shifted against each other along the [111] direction^17^, connected to a transverse optical phonon softening^30^. Plekhanov et al.^24^ tested small displacements and theoretically predicted that for a certain range of displacements a Z_2_ topological insulator can exist.

In general, all IV–VI compounds are close to such a structural phase transition due to their mixed covalent-ionic bonding. They belong to the family of 10 electron systems and crystallize either in the cubic rock salt, rhombohedral, or orthorhombic structure. As calculated by Littlewood^31, 32^, the type of structure in which a IV–VI compound crystallizes critically depends on the values of two bond orbital coordinates—one is a measure of the ionicity and the other of covalency or *s–p* hybridization, and based on these a phase diagram has been established^31, 32^. Due to the not fully saturated *p*-bonds, the rock salt structure is inherently unstable against rhombohedral distortions^31–33^, which reduces the six nearest neighbors to three. The cubic/rhombohedral phase boundary is determined by the electronegativity difference between the constituting elements and as a physical explanation of the instability the resonating bond model was invoked^34^. For the six nearest neighbors, the number of available *p*-electrons (six per atom pair) is not sufficient to stabilize the cubic bonds^35^. Thus, SnTe and GeTe assume a rhombohedral structure and even for PbTe minute addition of <0.5% of Ge suffices to drive Pb_1-*x*_Ge_*x*_Te into the rhombohedral phase, rendering it ferroelectric^36, 37^. Thus, very small changes on the group IV lattice sites give rise to structural phase transitions. Indeed, for our Bi-doped films, we find a small Bi-induced rhombohedral distortion along the [111] direction using x-ray diffraction (Supplementary Figs. 4–6 and Supplementary Note 4). This indicates a ferroelectric inversion symmetry breaking as the underlying physical mechanism, which has been suggested to be much enhanced at the surface^21, 38^. Because of the (111) orientation of our films, this symmetry breaking lifts the even number degeneracy of the Dirac cones such that a gap is opened only at the $\bar \Gamma$ point. This leaves an odd number of Dirac cones intact at the three ${\bar {\rm M}}$ points, causing the topological phase transition.

Finally, we would like to address the role of Bi as decisive ingredient for symmetry breaking even at small concentrations. Bi is another 10 electron system^33^, covalently bonded and crystallizing in a rhombohedral structure. Accordingly, when incorporated at group IV lattice sites in Pb_1−*x*_Sn_*x*_Se, it shifts the alloy toward a more covalently bonded structure in the phase diagram^31, 32, 39^. Moreover, on such lattice sites, Bi also reduces the cation vacancy concentration and the latter strongly enhances the ferroelectric Curie temperature *T*_C_ of the cubic-to-rhombohedral phase transition^30, 35, 38, 40^. Indeed, in SnTe thin films with reduced vacancy concentration, ferroelectricity up to room temperature was recently reported^38^. Thus, both effects of the Bi, higher covalency, and lower vacancy concentration, contribute to structural symmetry breaking and trigger the novel TCI-to-Z_2_ topological phase transition discovered in the present work.

## Methods

### Sample growth and characterization

Epitaxial growth of (111) Pb_1−*x*_Sn_*x*_Se films on BaF_2_ substrates was performed using molecular beam epitaxy (MBE) in ultrahigh vacuum conditions better than 5 × 10^−10^ mbar at a substrate temperature of 380 °C. Effusion cells filled with stoichiometric PbSe and SnSe were used as source materials, as well as a ternary Pb_1−*x*_Sn_*x*_Se source with *x*_Sn_ = 25%. Bi-doping was realized using a Bi_2_Se_3_ effusion cell. The chemical composition of the layers was varied over a wide range from *x*_Sn_ = 0 to 40% by control of the SnSe/PbSe beam flux ratio, and a two-dimensional growth was observed by in situ reflection high-energy electron diffraction (Supplementary Fig. 1). The film thickness was in the range of 1–3 µm.

### High-resolution ARPES

For the ARPES measurements, the films were capped in situ in the MBE chamber with a 200 nm thick amorphous Se layer at room temperature to protect the surface against oxidation during transport to the BESSY II synchrotron radiation source in Berlin, Germany. There the Se cap was completely desorbed in the ARPES preparation chamber by annealing at about 230 °C for 15 min in 3 × 10^−10^ mbar. ARPES measurements were performed at the UE112-PGM2a beamline of BESSY II at pressures better than 1 × 10^−10^ mbar using linearly polarized *p* + *s* photons incident on the sample under an angle *ϕ* = 45°. We used photon energies between 18 and 23 eV for the temperature-dependent ARPES measurements of the band dispersions, and a photon energy of 90 eV for the core levels (see Supplementary Fig. 12 and Supplementary Note 10). Emitted photoelectrons were detected with a Scienta R8000 electron energy analyzer at the ARPES 1^2^ endstation. Overall resolutions of the ARPES measurements were 5 meV (energy) and 0.3° (angular).

### Composition and structural characterization

The composition of the epilayers was determined using high-resolution x-ray diffraction and the Vegard’s law (Supplementary Fig. 2 and Supplementary Note 2). We employed a Seifert diffractometer equipped with primary and secondary monochromator crystals. Upon Bi incorporation, the rhombohedral lattice distortion was determined from reciprocal space maps recorded around the (513) reflection (see Supplementary Figs. 4–6 and Supplementary Note 4).

### Electrical characterization

The dopant concentration and transport properties were assessed by Hall effect measurements at 77 K (Supplementary Fig. 3 and Supplementary Note 3), evidencing carrier mobilities as high as 10^4^ cm^2^ V^−1^ s^−1^ in dependence of the carrier concentration.

### Data availability

The authors declare that all data supporting the findings of this study are available within the paper and its Supplementary Information files.

## Electronic supplementary material

Supplementary Information

## References

[1] Hasan MZ, Kane CL (2010). Colloquium: topological insulators. Rev. Mod. Phys..

[2] Ezawa M, Tanaka Y, Nagaosa N (2013). Topological phase transition without gap closing. Sci. Rep..

[3] Qi X-L, Zhang S-C (2011). Topological insulators and superconductors. Rev. Mod. Phys..

[4] Bernevig, B. A. & Hughes, T. L. *Topological Insulators and Topological Superconductors* (Princeton University Press, Princeton, 2013).

[5] Fu L, Kane CL (2007). Topological insulators with inversion symmetry. Phys. Rev. B.

[6] Fu L, Kane CL, Mele EJ (2007). Topological insulators in three dimensions. Phys. Rev. Lett..

[7] Brahlek M (2012). Topological-metal to band-insulator transition in (Bi_1−*x*_In_*x*_)_2_Se_3_ thin Films. Phys. Rev. Lett..

[8] Wu L (2013). A sudden collapse in the transport lifetime across the topological phase transition in (Bi_1−*x*_In_*x*_)_2_Se_3_. Nat. Phys..

[9] Xu S-Y (2011). Topological phase transition and texture inversion in a tunable topological insulator. Science.

[10] Fu L (2011). Topological crystalline insulators. Phys. Rev. Lett..

[11] Hsieh TH (2012). Topological crystalline insulators in the SnTe material class. Nat. Commun..

[12] Tanaka Y (2012). Experimental realization of a topological crystalline insulator in SnTe. Nat. Phys..

[13] Xu S-Y (2012). Observation of a topological crystalline insulator phase and topological phase transition in Pb_1-*x*_Sn_*x*_Te. Nat. Commun..

[14] Ando Y, Fu L (2015). Topological crystalline insulators and topological superconductors: from concepts to materials. Annu. Rev. Condens. Matter Phys..

[15] Dziawa P (2012). Topological crystalline insulator states in Pb_1-*x*_Sn_*x*_Se. Nat. Mater..

[16] Wuttig M, Yamada N (2007). Phase-change materials for rewriteable data storage. Nat. Mater..

[17] Schmitte, F. J. in *Physics of Non-Tetrahedrally Bonded Binary Compounds II* (ed. Madelung, O.) Landolt-Börnstein, New Series III/17F (Springer, Berlin, 1983).

[18] Chattopadhyay T, Boucherle JX, von Schnering HG (1987). Neutron diffraction study on the structural phase transition in GeTe. J. Phys. C.

[19] Di Sante D, Barone P, Bertacco R, Picozzi S (2013). Electric control of the giant Rashba effect in bulk GeTe. Adv. Mater..

[20] Okada Y (2013). Observation of Dirac node formation and mass acquisition in a topological crystalline insulator. Science.

[21] Zeljkovic I (2015). Dirac mass generation from crystal symmetry breaking on the surfaces of topological crystalline insulators. Nat. Mater..

[22] Wojek BM (2015). Direct observation and temperature control of the surface Dirac gap in a topological crystalline insulator. Nat. Commun..

[23] Niu C (2015). Two-dimensional topological crystalline insulator and topological phase transition in TlSe and TlS monolayers. Nano. Lett..

[24] Plekhanov E, Barone P, Di Sante D, Picozzi S (2014). Engineering relativistic effects in ferroelectric SnTe. Phys. Rev. B.

[25] Safaei S, Galicka M, Kacman P, Buczko R (2015). Quantum spin hall effect in IV-VI topological crystalline insulators. New J. Phys..

[26] Neupane M (2015). Topological phase diagram and saddle point singularity in a tunable topological crystalline insulator. Phys. Rev. B.

[27] Polley CM (2014). Observation of topological crystalline insulator surface states on (111)-oriented Pb_1−*x*_Sn_*x*_Se films. Phys. Rev. B.

[28] Strauss AJ (1967). Inversion of conduction and valence bands in Pb_1-*x*_Sn_*x*_Se alloys. Phys. Rev..

[29] Zykov VA, Gavrikova TA, Il’in VI, Nemov SA, Savintsev PV (2001). Effect of bismuth impurity on carrier density in PbSe:Bi:Se epitaxial layers. Semiconductors.

[30] Sugai S (1977). Carrier density dependence of soft TO-phonon in SnTe by Raman scattering. Solid State Commun..

[31] Littlewood PB (1980). The crystal structure of IV-VI compounds. I. Classification and description. J. Phys. C Solid State Phys..

[32] Littlewood PB (1980). The crystal structure of IV-VI compounds. II. A microscopic model for cubic/rhombohedral materials. J. Phys. C Solid State Phys..

[33] Littlewood PB (1983). Structure and bonding in narrow gap semiconductors. Crit. Rev. Solid State Mater. Sci..

[34] Nimtz, G. & Schlicht, B. in *Springer Tracts in Modern Physics* 1–117, Vol. 98, Narrow-gap semiconductors (Springer, Berlin, 1983).

[35] Jantsch, W., Bussmann-Holder, A., Bilz, H. & Vogel, P. in *Springer Tracts in Modern Physics* 1-98, Vol. 99, Dynamical properties of IV-VI compounds (Springer, Berlin, 1983).

[36] Bangert E, Bauer G, Fantner EJ, Pascher H (1985). Magneto-optical investigations of phase-transition-induced band-structure changes of Pb_1-*x*_Ge_*x*_Te. Phys. Rev. B.

[37] Lebedev AI, Sluchinskaya IA (1995). Influence of random fields on the ferroelectric phase transition in IV–VI semiconductors. Ferroelectrics.

[38] Chang K (2016). Discovery of robust in-plane ferroelectricity in atomic-thick SnTe. Science.

[39] Kool BJ, Noheda B (2016). Ferroelectric chalcogenides–materials at the edge. Science.

[40] Iizumi M, Hamaguchi Y, Komatsubara KF, Kato Y (1975). Phase transition in SnTe with low carrier concentration. J. Phys. Soc. Jpn..

