# Supplementary material for: Topological quantum phase transition from mirror to time reversal symmetry protected topological insulator
Source: Nat Commun. 2017 Oct 17;8:968. doi: 10.1038/s41467-017-01204-0 (PMC5645419; doi:10.1038/s41467-017-01204-0)
Supplement: Supplementary file 1 — Supplementary Information [file 41467_2017_1204_MOESM1_ESM.pdf]

Supplementary Figures

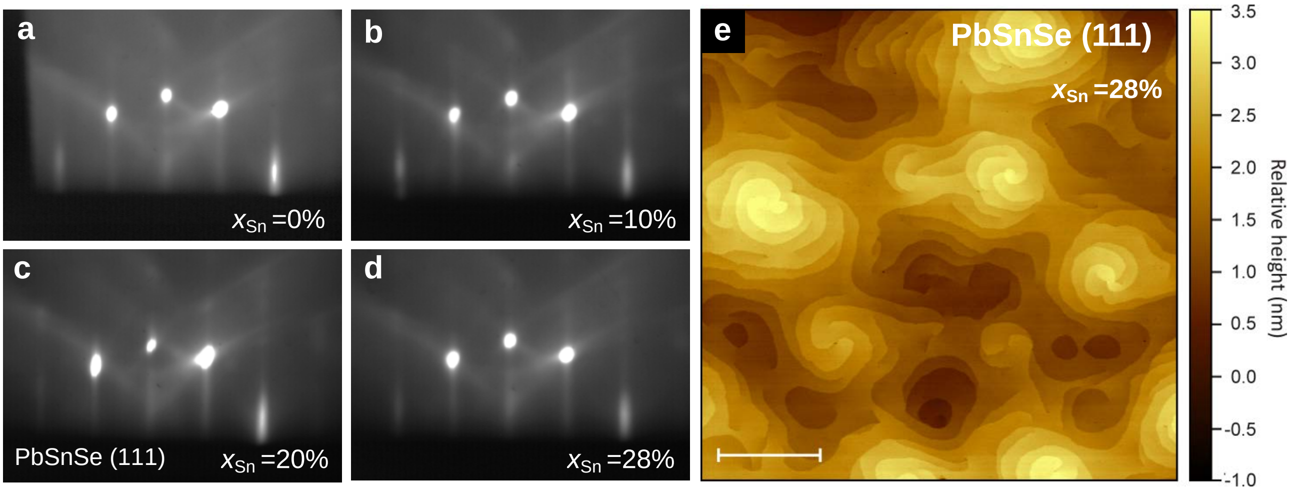

Supplementary Figure 1: **Growth and Surface Characterization.** Reflection high-energy electron diffraction of $Pb_{1-x}Sn_xSe$ epilayers on BaF_2 (111) substrates recorded during MBE growth for x_{sn} = 0, 10, 20 and 28% from (**a**) to (**d**), respectively. (**e**) Atomic force microscopy image of the x_{sn} = 28% epilayer with 1 µm thickness. Horizontal scale bar, 1 µm (white-solid line).

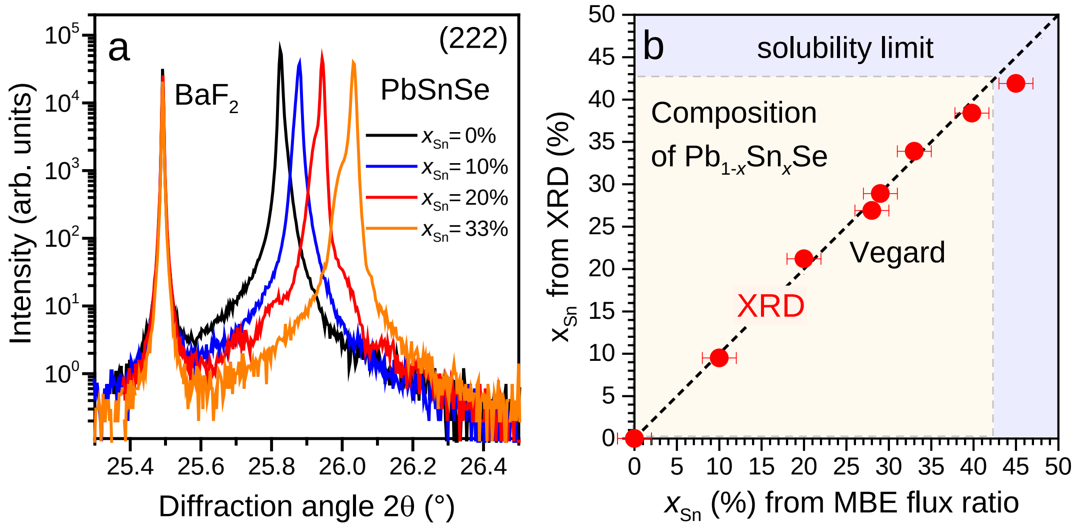

Supplementary Figure 2: **X-ray diffraction of $Pb_{1-x}Sn_xSe$ epilayers on BaF_2 (111).** (**a**) Radial (222) diffraction scans for selected compositions varying from x_{Sn} = 0 to 33%. (**b**) Sn content derived from Vegard's law (dashed line) plotted versus the beam flux ratio of SnSe/(PbSe+SnSe) used for molecular beam epitaxy (MBE). The boundary of the Sn solubility limit in single phase cubic $Pb_{1-x}Sn_xSe$ of about 42% is indicated. The error in x_{Sn} was derived from the 2% precision of the MBE flux measurements using the microbalance technique.

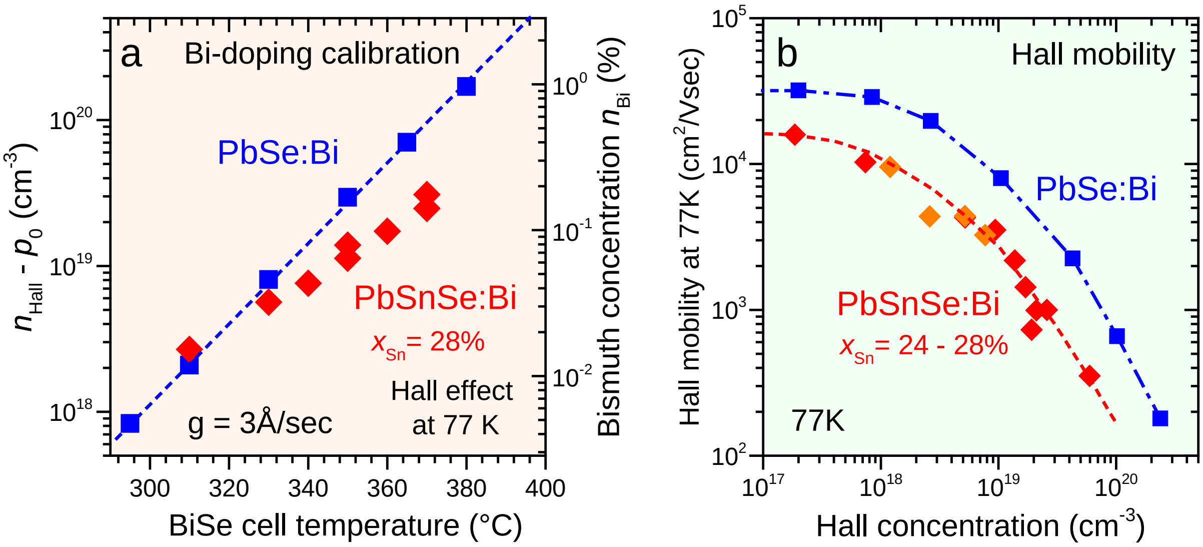

Supplementary Figure 3: **Bi doping and Hall effect transport measurements.** The measurements are obtained at 77 K for PbSe:Bi (■) and PbSnSe:Bi with x_{Sn} = 28% (◆). (**a**) Effective electron concentration ($n_{Hall} - p_0$) versus Bi_2Se_3 effusion cell temperature used during growth by molecular beam epitaxy for a constant film deposition rate of 3 Ås^{-1}. The background carrier concentration of undoped *p*-type reference layers due to cation vacancies was $p_0 = +2\times10^{17}$ for PbSe and $+8\times10^{17}$ cm^{-3} for $Pb_{0.72}Sn_{0.28}Se$. The dashed line represents the values expected for unity doping efficiency. The lower carrier concentration observed for $Pb_{0.72}Sn_{0.28}Se$ indicates a reduction of the doping efficiency with increasing Bi content, contrary to the behavior of PbSe. (**b**) Hall mobility of the epilayers versus carrier concentration. For low carrier concentrations, a saturation mobility of μ_{77K} = 32000 and 10500 cm^{2}V^{-1}s^{-1} is obtained for PbSe and $Pb_{0.72}Sn_{0.28}Se$, respectively.

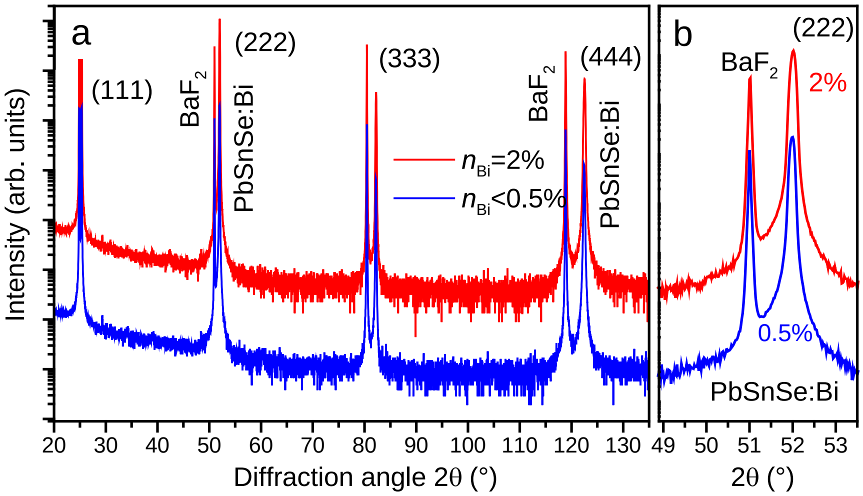

Supplementary Figure S4: **Structural effect of Bi-doping of $Pb_{1-x}Sn_xSe$.** (**a**) X-ray diffraction spectra of two $Pb_{0.72}Sn_{0.28}Se$ layers with high and low Bi dopant concentration (blue: n_{Bi} <0.5%, red: n_{Bi} = 2.2%), evidencing a single phase structure without any traces of secondary phases. (**b**) Zoom-in on the diffraction curves around the (222) Bragg reflection, evidencing no structural degradation due to Bi incorporation.

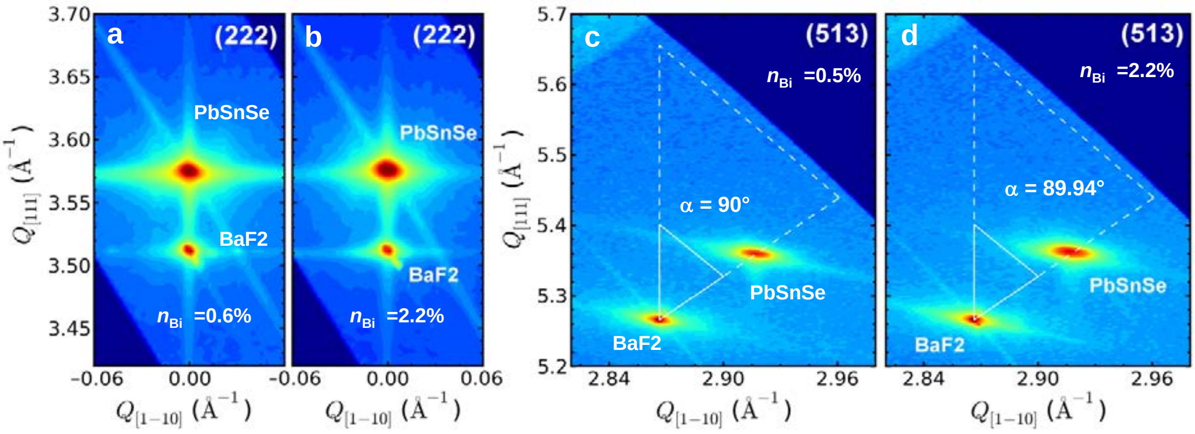

Supplementary Figure 5: **X-ray diffraction reciprocal space maps.** Results of two $Pb_{0.72}Sn_{0.28}Se$:Bi samples doped with n_{Bi} ~ 0.6% (**a, c**) and n_{Bi} = 2.2% (**b, d**), indicating a small rhombohedral lattice distortion for the latter with the distortion angle α = 89.94°. The reciprocal space maps were recorded around the symmetric (222) and asymmetric (513) reciprocal lattice points at room temperature.

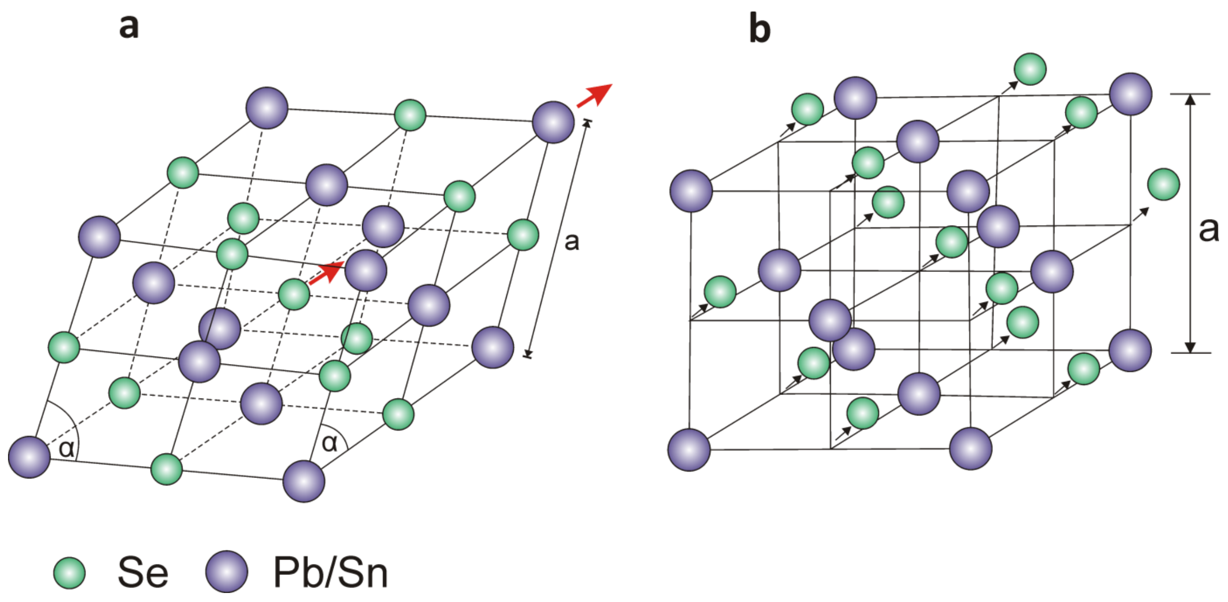

Supplementary Figure 6: **Rhombohedral distortion.** (**a**) Sketch of a rhombohedral distortion with rhombohedral angle α < 90°. This angle corresponds to an elongation along [111] direction. (**b**) A sublattice shift (green atoms) is shown which is responsible for ferroelectricity. For simplicity, this is shown for a cubic structure. The sublattice shift typically also leads to a rhombohedral distortion.

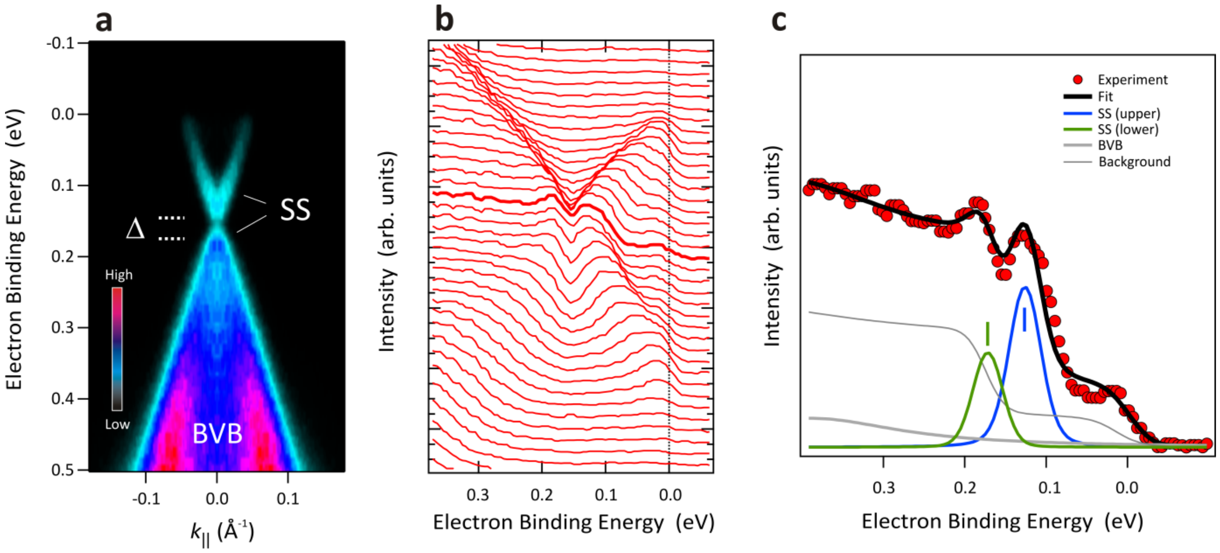

Supplementary Figure 7: **Determination of the surface band gap.** (**a**) Energy-momentum ARPES dispersion measured at 30 K and 18 eV photon energy for $Pb_{0.72}Sn_{0.28}Se$ doped with 0.6% Bi. (**b**) Corresponding energy-distribution curves (EDCs) extracted from **a**. The EDC at zero momentum is highlighted by a thick red solid line, and contains a double peak structure which is the signature of a ~45±10 meV gap. (**d**) Corresponding fit results (black solid lines) of the EDC at zero momentum (red circles) considering a Shirley-like background [4]. The size of the surface band gap is determined from the energy separation between the fitted Lorentzian peaks shown in blue (green) color, which are located at the energy minimum (maximum) of the upper (lower) part of the surface state (SS). The Lorentzian peaks shown in gray color as a thick solid line represent the contribution from the bulk-valence band (BVB).

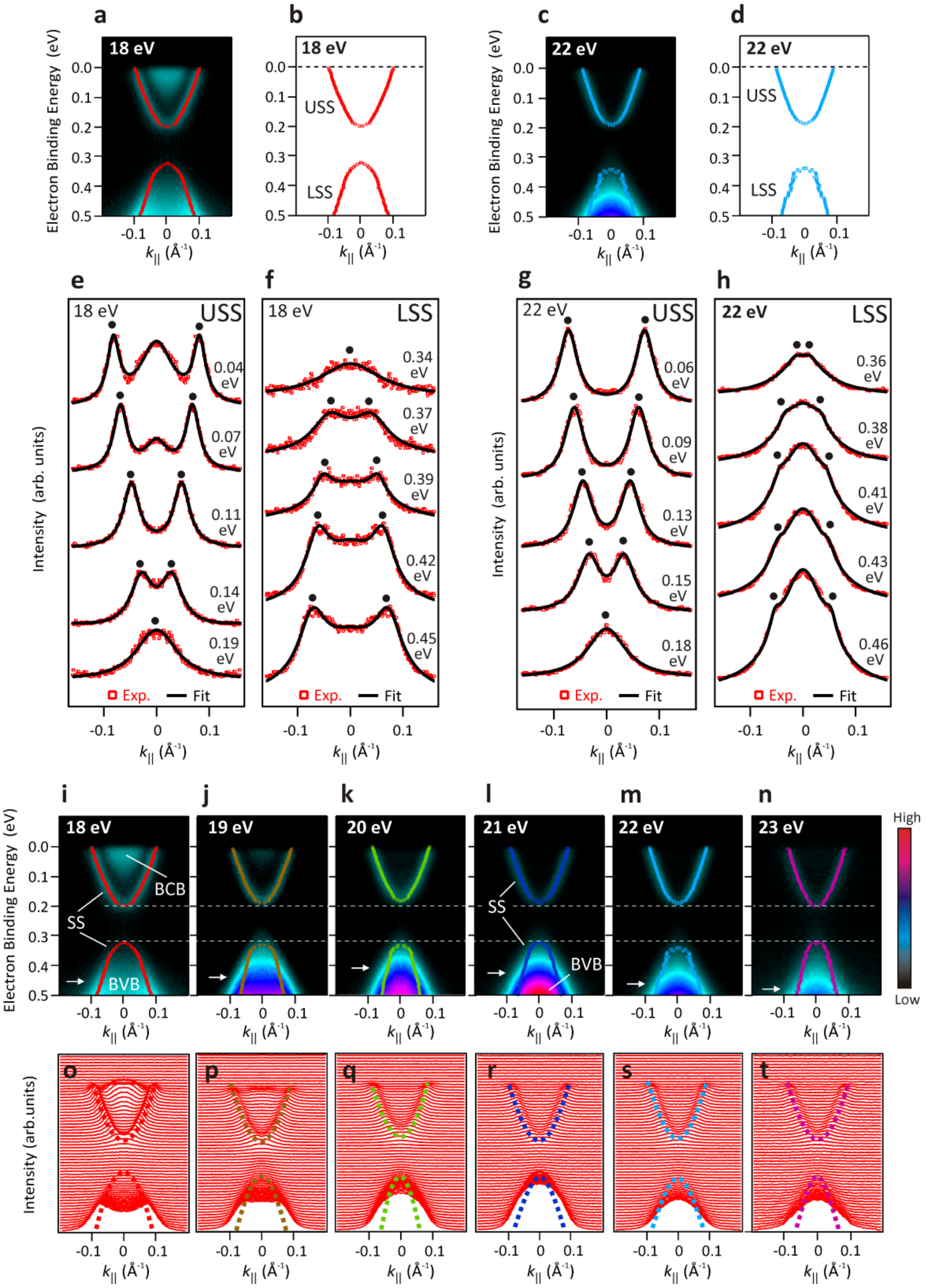

Supplementary Figure 8: **Photon energy dependence of $Pb_{0.72}Sn_{0.28}Se$ doped with 2.2% Bi.** (**a**) Band dispersion of the gapped surface state at 18 eV photon energy, with the peak positions extracted from fits to momentum-distribution curves (MDCs) superimposed. (**b**) Fit results as shown in (**a**), with the upper (USS) and lower (LSS) parts of the surface state indicated. (**c**) and (**d**): Analogous results for a photon energy of 22 eV as in (**a**) and (**b**), respectively. (**e-h**) Fits (black solid lines) to the experimental MDCs (red symbols) shown for selected binding energies across (**e**,**g**) the upper and (**f**,**h**) lower parts of the surface state at (**e**,**f**) 18 eV and (**g**,**h**) 22 eV photon energy. The intensity contributions from the surface state are marked with black filled circles on top of each fit. In (**h**), the intensity from the opposite branches of the surface state is clearly resolved as shoulders around the intensity of the bulk-valence band. (**i-n**) Photon-energy dependent ARPES spectra shown in Figs. 2h-m of the main text. For each photon energy, we have superimposed the fit results to MDCs shown in Fig. 2n of the main text. (**o-t**) MDCs extracted from the data shown in panels (**i-n**). Dashed lines are guides to the eye qualitatively following the fitted band dispersions.

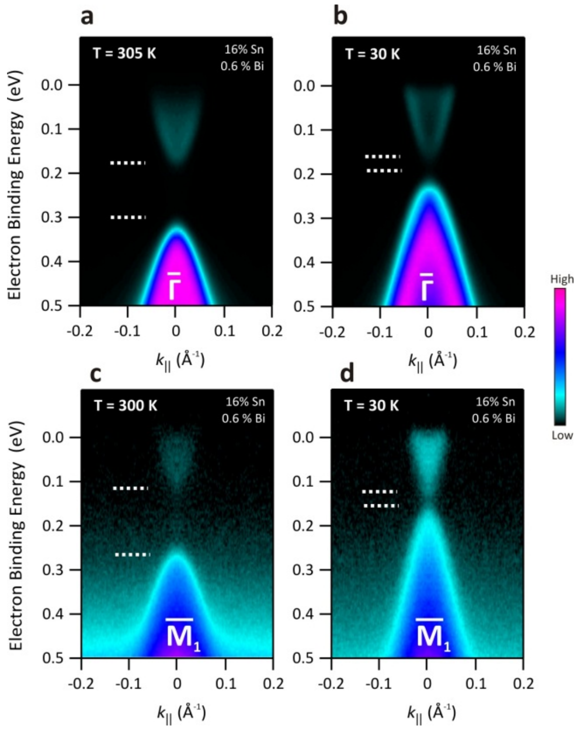

Supplementary Figure 9: **ARPES spectra of a control sample in the trivial state.** (**a-d**) Energy-momentum ARPES dispersions of a $Pb_{1-x}Sn_xSe$ (111) epilayer with 16% Sn and 0.6% Bi doping at (**a**,**b**) $\bar{\Gamma}$ and (**c**,**d**) $\bar{M}$, acquired at (**a**,**c**) room temperature and (**b**,**d**) 30 K. For this sample, the band gap becomes smaller but remains open at the lowest measured temperature. The data show that the band gaps at the $\bar{\Gamma}$ and $\bar{M}$ points exhibit similar behavior with decreasing temperature.

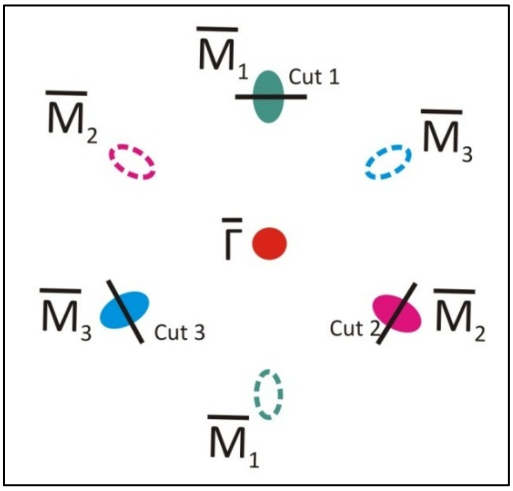

Supplementary Figure 10: **Schematic of the surface Brillouin zone of $Pb_{1-x}Sn_xSe$ (111).** The energy-momentum ARPES dispersions around the $\overline{M}$ points have been acquired with $k_{\parallel}$ running perpendicular to each individual $\overline{\Gamma}\overline{M}$ direction as indicated by the black solid lines.

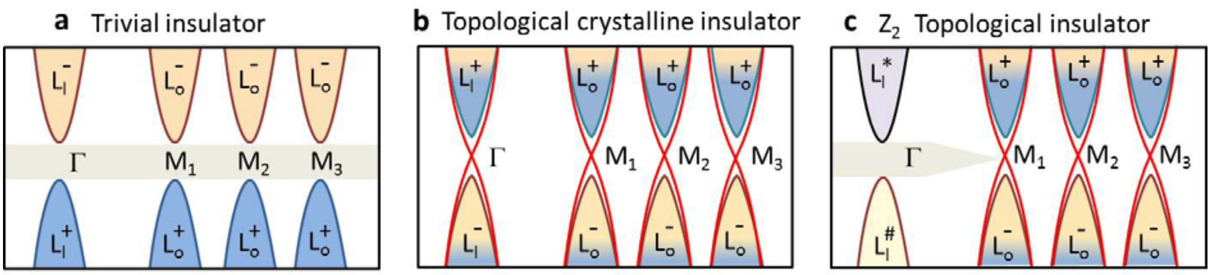

Supplementary Figure 11: **Sketch of the different topological phases.** (**a-c**) Relative position of the band extrema and Dirac cones at the $\overline{\mathrm{M}}$ and $\overline{\Gamma}$ points of $Pb_{1-x}Sn_xSe$ (111) in the (**a**) trivial insulator, (**b**) topological crystalline insulator (TCI), and (**c**) Z_2 topological insulator phases. The surface state Dirac cones (red lines) together with the bulk conduction and valence bands are shown. In the trivial state, all gaps at $\overline{\mathrm{M}}$ and $\overline{\Gamma}$ are equal. In the TCI state the bulk bands are inverted and a topological surface state is formed at all four symmetry points. In the Z_2 topological insulator state, the Dirac cones are closed at all three $\overline{\mathrm{M}}$ points but open at $\overline{\Gamma}$. The reversal of the color of the bulk bands indicates the presence of the bulk band inversion.

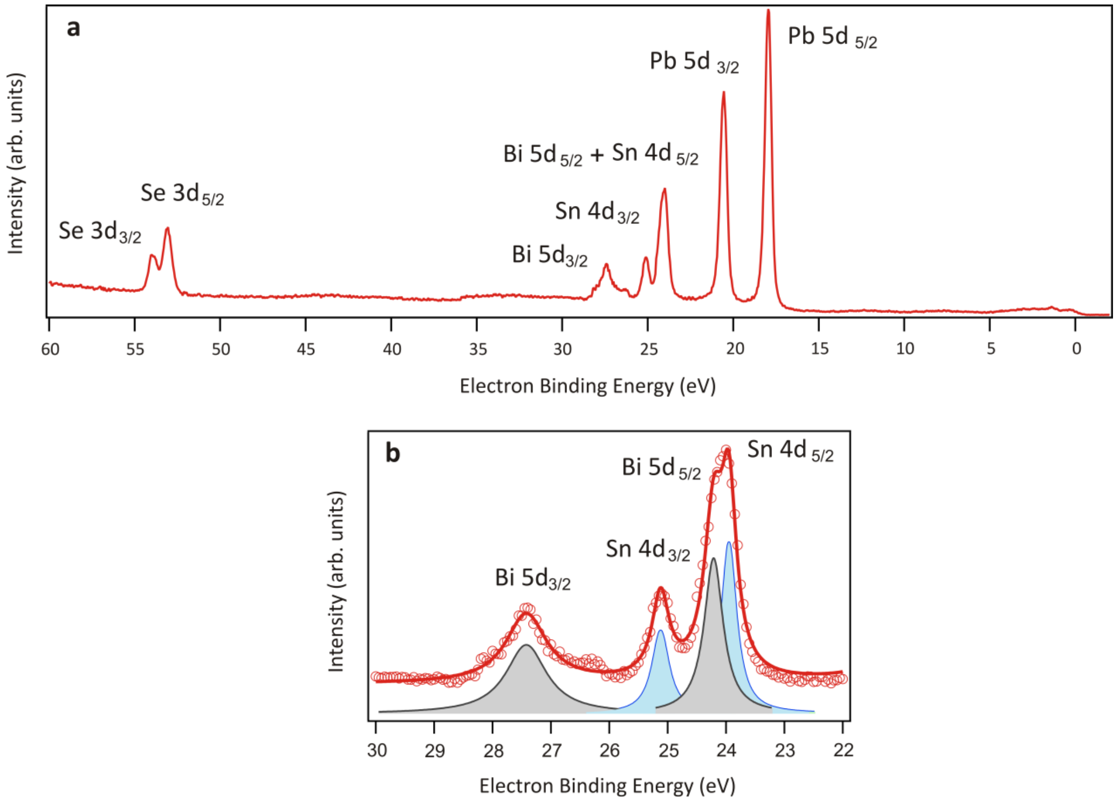

Supplementary Figure 12: **Core level spectra of $Pb_{0.72}Sn_{0.28}Se$:Bi film**. (**a**) 2.2% Bi doping measured after desorption of the Se cap at the ARPES setup. (**b**) Fitted spectra.

Supplementary Notes

Supplementary Note 1: Sample growth by molecular beam epitaxy

Epitaxial growth of (111) $Pb_{1-x}Sn_xSe$ films on BaF_2 substrates was performed in a Riber 1000 system for molecular beam epitaxy (MBE) in ultrahigh vacuum conditions better than 5×10^{-10} mbar. Effusion cells filled with stoichiometric PbSe and SnSe were used as beam flux sources. Alternatively, also a ternary $Pb_{0.75}Sn_{0.25}Se$ source was used. Bi-doping was realized using a compound Bi_2Se_3 effusion cell. The chemical composition of the layers was varied over a wide range from x_{Sn} = 0 to 40% by variation of the SnSe/PbSe beam flux ratio measured using a quartz microbalance moved into the substrate position. The composition of the layers determined from the beam flux ratio agrees within ±2% to the composition determined independently determined by x-ray diffraction as described below. The growth rates were around 1 µm h^{-1} (~1 monolayer s^{-1}) and the growth temperature was set to 380°C as checked by an IRCON infrared pyrometer. The film thickness was in the range of 1–3 µm.

For all layers smooth two-dimensional (2D) growth occurs after few nanometer deposition on BaF_2 (111) as evidenced by Supplementary Figs. 1a-d that present the reflection high-energy electron diffraction (RHEED) patterns recorded *in situ* during MBE growth for films with various Sn compositions. The high quality of the layers is evidenced by sharp diffraction spots on the Laue circle and intense Kikuchi lines arising from diffraction from subsurface bulk lattice planes. No surface reconstruction was observed during deposition. The surface of the films is atomically flat, exhibiting only single monolayer steps of 3.52 Å height as exemplified by the atomic force microscopy (AFM) image presented in Supplementary Fig. 1e for x_{sn} = 28%. At the given growth temperature, growth proceeds in a 2D step-flow mode. Due to pinning of surface steps at screw type threading dislocations originating from the $Pb_{1-x}Sn_xSe$ / BaF_2 (111) lattice-mismatch of $\Delta a/a$ ~ 1.6% a characteristic spiral step structure is formed (cf. Supplementary Fig. 1e) similar as described in Ref. [1].

Supplementary Note 2: X-Ray diffraction and composition

The structural properties and composition of the $Pb_{1-x}Sn_xSe$ epilayers were characterized in detail by high-resolution x-ray diffraction (XRD) using a Seifert diffractometer equipped with primary and secondary monochromator crystals. Films with thicknesses larger than 1 µm are generally fully relaxed as found by reciprocal space mapping of asymmetric Bragg reflections. With increasing Sn content, the diffraction peaks shift to larger diffraction angles as illustrated by Supplementary Fig. 2a for the (222) Bragg reflection, indicating a corresponding decrease of the lattice constant a_0. From the evaluation of the lattice parameter, we find that the lattice constant closely follows the Vegard's law given by:

$$a_0\,(x_{Sn}) = 6.124 - 0.123 \cdot x_{Sn}\ [\text{Å}] \qquad (1)$$

in agreement with previous works [2]. Here, a_0 = 6.124 Å is the lattice constant of pure PbSe. Due to the sharp diffraction peaks and the high-resolution x-ray diffraction set-up the precision of the lattice constant determination is ±0.001 Å, which translates into a precision for x_{Sn} of ±0.01, i.e., of ±1%. Supplementary Fig. 2b shows the resulting composition of the layers versus that obtained from *in situ* beam flux measurements using the quartz balance method, evidencing a very good agreement without adjustable parameters. Films with Sn content above 40% are found to be no longer single phase, resulting in a splitting of the diffraction peaks. This is due to the fact that SnSe exhibits an orthorhombic crystal structure, for which reason the solubility of Sn in cubic rock salt $Pb_{1-x}Sn_xSe$ is limited to about 42%.

Supplementary Note 3: Bi-doping and electrical characterization

Undoped $Pb_{1-x}Sn_xSe$ exhibits a *p*-type background carrier concentration due to cation (Pb/Sn) vacancies formed during growth. These cation vacancies form resonant acceptor like energy levels and thus induce a *p*-type hole conductivity. The *p*-type carrier concentration increases with increasing Sn content from around 10^{17} cm^{-3} for PbSe to above 10^{18} cm^{-3} for $x_{Sn} > 30\%$. Incorporating Bi makes the system *n*-type and allows tuning the Fermi level into the conduction band as required for studying the properties of the of the entire Dirac cones of the topological surface states by angle-resolved photoemission (ARPES). We use a Bi_2Se_3 effusion cell for doping, which promotes substitutional incorporation of Bi on cation lattice sites without the need of an additional Se flux. The solubility of Bi in PbSe and SnSe amounts to several percent according to the quasi binary phase diagrams [1], however, for *n*-doping only a small Bi_2Se_3 flux in the range of 10^{-5}-10^{-2} ML s^{-1} range is actually required, depending on the desired doping level. This doping flux was calibrated as described in detail in Ref. [3].

Doping action of Bi and the resulting electrical properties of the films were evaluated by Hall measurements of a large series of samples as shown in Supplementary Fig. 3. For Bi-doped PbSe the measured electron concentration n_H corrected for the background hole concentration p_0 increases linearly with Bi flux and therefore changes exponentially with Bi_2Se_3 effusion cell temperature (*cf.* Supplementary Fig. 3a, ■), indicating a unity doping efficiency in agreement with previous results for Bi-doped PbTe films [3]. This therefore provides a reliable calibration of Bi concentrations within a relative error of 10%.

For $Pb_{1-x}Sn_xSe$ films, the electron concentration n_H - p_0 (Supplementary Fig. 3b, ◆) is generally found to be lower for the same growth conditions. This indicates a reduced doping efficiency of Bi in PbSnSe compared to that in PbSe, in particular at higher doping concentrations. As shown in Supplementary Fig. 3b, for small electron densities in the 10^{17} cm^{-3} range, the mobility of the epilayers at 77K is as high as $\mu_{77K} = 32000$ cm^2 $V^{-1}s^{-1}$ for PbSe and 10.500 cm^2 $V^{-1}s^{-1}$ for $Pb_{0.72}Sn_{0.28}Se$. Due to the increased scattering the mobility rapidly decreases with increasing Bi-content in particular above the 10^{18} cm^{-3} level, as we have also previously reported for PbTe [3].

Supplementary Note 4: Structural effect of Bi-doping

The influence of Bi-doping on the structural properties of the epilayers was evaluated by high-resolution x-ray diffraction as shown by Supplementary Fig. 4. For Bi-concentrations as high as 2.2% (doping concentration of 3.5×10^{20} cm^{-3}) we do not find any indication of phase separation or secondary phase formation and no broadening of the diffraction peaks occurs (Supplementary Fig. 4b).

For further analysis of possible lattice deformations, reciprocal space maps were recorded around the (222) and (513) reciprocal lattice points as shown in Supplementary Fig. 5 for two $Pb_{0.72}Sn_{0.28}Se$ epilayers with high (2.2%) and low (<0.5%) Bi-doping. From the fit of the peak positions, the in-plane and out-of-plane lattice constants, as well as unit cell corner angle α (rhombohedral distortion) was derived using the relation:

$$\sin(\alpha/2) = a_{//}\sqrt{3}\,(2a_{\perp}^2 + 4\,a_{//}^2)^{-1/2} \qquad (2)$$

For low Bi-concentration the layers exhibit a cubic lattice structure with a corner angle α = 90°. The experimental error in α is of ±0.01°, as derived from the errors in the lattice parameter determination (±0.001 Å). For the high-Bi doped layers, however, a rhombohedral lattice distortion is found with α ~ 89.94° for $n_{Bi} = 2.2\%$ as shown by Supplementary Fig. 5d. This indicates that Bi-doping modifies the lattice structure and thus impacts the topological surface state in the presence of a rhombohedral distortion. The visualization of the rhombohedral angle α and the shift of anion and cation (111) planes that typically cause the

rhombohedral distortion is depicted in Supplementary Fig. 6. On the other hand, we point out that for the sample with n_{Bi} = 1%, since the distortion is still too weak, from the XRD data we cannot conclusively derive the exact point of the structural phase transition considering the error bars.

Supplementary Note 5: Angle-resolved photoemission spectroscopy

Photoemission experiments were performed at the undulator beamline UE112-PGM2a of the BESSY II synchrotron radiation source in Berlin, Germany. We used the endstation ARPES 1[2] which is equipped with a Scienta R8000 hemispherical analyzer allowing to detect emitted photoelectrons up to acceptance angles of 30°. The base pressure during experiments was better than 1×10^{-10} mbar. The epitaxial films were capped *in situ* after MBE growth with a 200 nm thick amorphous Se layer at room temperature to protect the surface against oxidation during transport to the ARPES setup, where the Se cap was completely desorbed in the preparation chamber by annealing at about 230°C for 15 min in 3×10^{-10} mbar. The corresponding valence band ARPES data were collected in a wide range of temperatures using linearly-polarized *p*+*s* photons with energies ranging from 18 to 23 eV. The ARPES dispersions around the $\overline{\Gamma}$ and $\overline{\mathrm{M}}$ points were acquired using the photon beam geometry shown in Fig. 1a of the main text, where the light is incident on the sample under and angle of ϕ=45° with respect to the surface normal. The light incidence and electron detection planes were parallel to the $\overline{\mathrm{M}}$-$\overline{\Gamma}$-$\overline{\mathrm{M}}$ and $\overline{\mathrm{K}}$-$\overline{\Gamma}$-$\overline{\mathrm{K}}$ high symmetry directions of the surface Brillouin zone, respectively. The corresponding energy and angular resolutions were set to 5 meV and 0.1°, respectively.

Supplementary Note 6: Determination of the surface band gap

To determine the size of the surface band gaps as a function of Bi and Sn concentrations as well as of temperature, we have fitted the experimental energy-distributions curves (EDCs) at zero momentum extracted from the ARPES spectra shown in the main text. In Supplementary Fig. 7, we summarize the procedure used by taking a $Pb_{0.72}Sn_{0.28}Se$ sample doped with 0.6% Bi as an example. Supplementary Fig. 7a displays the corresponding energy-momentum ARPES dispersion measured at 30 K and 18 eV photon energy. The intensity contributions from the bulk-valence band (BVB) states at high binding energies are clearly distinguishable from the dispersion of the upper and lower part of the surface state (SS) at lower binding energies. In particular, we observe that in contrast to the undoped $Pb_{0.72}Sn_{0.28}Se$ sample measured under the same experimental conditions (see Fig. 2a of the main text), incorporating Bi leads to an intensity dip at the energy position of the original Dirac point which is the signature of a small gap. We also note that by increasing the Bi concentration, as also seen in Figs. 2c,d of the main text, the intensity dip becomes more and more pronounced which is related to the widening of the surface gap.

In Supplementary Fig. 7b we show the corresponding EDCs extracted from Supplementary Fig. 7a, where the EDC at normal emission is highlighted with a thick red line. The corresponding fit results (black solid lines) of the EDC (red circles) are shown in Supplementary Fig. 7c. The error bars in the determination of the surface gap shown in the main text correspond to the uncertainty of determining the energy position of the band dispersions, and are estimated from the standard deviations of the peak positions over several fitting cycles. Specifically, the size of the surface band gap is determined from the energy separation between the fitted Lorentzian peaks shown in blue (green) color, which are located at the energy minimum (maximum) of the upper (lower) part of the SS. We also point out that a lower limit of the surface band gap can be obtained from the ARPES spectra after considering the contribution from the linewidth broadening. The lower limit of the surface

band gap is approximately represented by horizontal dashed lines around the region of the gap in Figs. 2a-d and Fig. 3 of the main text. In Supplementary Figs. 7c, other Lorentzian peaks shown in gray color are contributions from the bulk-valence band (BVB). To extract the energy positions, the experimental ARPES spectra were fitted by a sum of Lorentzian functions plus a background. A typical spectrum containing N number of peaks was fitted by a function involving a convolution of the form:

$$\mathrm{I}(E,k) = \left[\mathrm{f}(E,T)\cdot\sum_{i=1}^{\mathrm{N}}\mathrm{M}_i^2\cdot\mathrm{A}_i(E_i,w_i)+\mathrm{B}(E)\right]\otimes\mathrm{G}(E) \qquad (3)$$

Where E_i, ω_i, and the matrix elements M_i are fitting parameters corresponding to the binding energy, width, and intensity of each Lorentzian peak, and f(E,T) is the Fermi function. The spectral function $\mathrm{A}_i(E_i,\omega_i)$ is approximated by Lorentzian functions, and B(E) is assumed to be a Shirley-like background [4]. The full width at half maximum (FWHM) of the Gaussian slit function G(E) corresponds to the total energy resolution of the experiment, which is photon-energy dependent.

Supplementary Note 7: Photon energy dependence of $Pb_{0.72}Sn_{0.28}Se$ doped with 2.2% Bi

To analyze the energy-momentum band dispersions of the surface state as a function of photon energy, we have fitted the experimental momentum-distribution curves (MDCs) extracted from the ARPES measurements shown in Fig. 2h-m of the main text. In Supplementary Figs. 8a-h, we summarize the procedure used by taking representative results at 18 eV and 22 eV photon energy as an example. For the fitting procedure, we used a sum of Lorentzian peaks plus a constant background convoluted with a Gaussian function representing the momentum resolution, and thus a fit function which is similar to Supplementary Eq. (3) but with the electron momentum parallel to the surface as the main variable. Supplementary Fig. 8a shows the fit results of the upper and lower parts of the surface state superimposed on the corresponding ARPES dispersion measured at 18 eV. The fitted peak positions are also shown independently in Supplementary Fig. 8b. Analogous results for measurements at 22 eV photon energy are displayed in Supplementary Figs. 8c,d. From this comparison, we observe good agreement concerning both the energy positions of the upper and lower parts of the surface state as well as their overall dispersion with momentum parallel to the surface.

In Supplementary Figs. 8e-h, we show few-selected fits (black solid lines) to experimental MDCs (red symbols) obtained at various binding energies across the upper and lower parts of the surface state for photon energies of 18 eV (Supplementary Figs. 8e,f) and 22 eV (Supplementary Figs. 8g,h). The intensity contributions from the surface state are marked with black filled circles on top of each fit. Additional intensities from the bulk-conduction band (BCB) near the Fermi level and from the bulk-valence band (BVB) at high binding energies were fitted by extra Lorentzian peaks introduced into the analysis procedure. As seen in Supplementary Figs. 8e-h, the obtained fits are in remarkable agreement with the experimental MDCs. The intensity contributions from the upper part of the surface state (Supplementary Figs. 8e,g) appear as distinct peaks in the corresponding MDCs. This is also the case for the whole measured photon-energy range shown in Figs. 2h-m of the main text, even for photon energies where the dispersion of the BCB is clearly observed. Similarly, as seen in Supplementary Fig. 8f, the lower part of the surface state appears as pronounced peaks around the BVB intensity, which becomes progressively smaller with decreasing binding energy. This situation is more or less representative for fits to experimental MDCs up to photon energies of 21 eV. At higher photon energies, despite the dispersion of the BVB with momentum perpendicular to the surface k_z, we clearly resolve the opposite branches of the lower part of the surface state as pronounced shoulders around the BVB intensity (see

Supplementary Fig. 8h). This allows us to extract the energy positions of the lower surface state with relatively good accuracy, despite the partial overlap with the BVB which nevertheless introduces additional errors in the fitted band dispersions. The error bars in the MDC fits were estimated from the standard deviations of the peak positions over several fitting cycles. In particular, the error bars in $\Delta k_{||}$ (ΔE) shown in Fig. 2n of the main text for each photon energy were taken as the maximum error obtained for the whole fitted range, and the corresponding error bars in energy were estimated from the obtained $\Delta k_{||}$ values. These error bars represent the maximum uncertainty in determining the corresponding band dispersions as extracted from the MDC fits. Supplementary Figs. 8i-n display the photon-energy dependent ARPES spectra shown in Figs. 2h-m of the main text, where we have superimposed the corresponding results of the MDC fits for each photon energy. By comparing the maximum error bar obtained from the MDC fits to the maximum deviation between the fitted results for different photon energies shown in Fig. 2n of the main text, we derive a total accuracy representing the upper bound for the maximal k_z dispersion of the surface state of ±20 meV. This result strongly indicates that the gapped surface state is two dimensional, in contrast to the three-dimensional character of the BCB or the BVB which clearly disperse with photon energy as seen in Supplementary Figs. 8i-n.

Supplementary Note 8: ARPES of the surface state in the topologically trivial state

To determine whether in the topologically trivial phase the temperature dependence of the band gaps is similar at the $\bar{\Gamma}$ and at $\bar{M}$ points, ARPES data were recorded for a ~0.6% Bi-doped $Pb_{1-x}Sn_xSe$ sample with a Sn content of 16% at temperatures ranging from 300 to 30 K. This particular Sn content was chosen to be close to the quantum critical point of the phase transition, but staying in the trivial phase for all investigated temperatures. The ARPES data shown in Supplementary Fig. 9 reveal both the valence and conduction band states with an open gap corresponding to the non-inverted band structure. At $\bar{\Gamma}$, we clearly observe the contribution from topologically trivial surface states forming as precursor states of the quantum-phase transition, which are also seen in Figs. 1-3 of the main text in agreement with previous observations on undoped topological crystalline insulators [5]. We also point out that the same precursor states have been observed in quantum-phase transitions between trivial and Z_2 topological insulators, which in addition preserve the helical spin texture despite their trivial origin [6]. Note that the intensity from the precursor states at the $\bar{M}$ points is less resolved in our data most probably because of their tilted projection onto the (111) plane, which corresponds to an electron emission angle of about 21° in the particular geometry shown in Supplementary Fig. 10.

Supplementary Note 9: Band gaps and Dirac cones in the different topological phases

As described in the main text, $Pb_{1-x}Sn_xSe$ (111) exists in different topological states depending on temperature, Sn and Bi content. For low x_{Sn} < 16%, $Pb_{1-x}Sn_xSe$ is topologically trivial with an open and equal band gap at the $\bar{M}$ and $\bar{\Gamma}$ points of the surface Brillouin zone as shown schematically in Supplementary Fig. 11a. For higher x_{Sn}, a bulk band inversion occurs at low temperatures, which renders $Pb_{1-x}Sn_xSe$ as topological crystalline insulator, in which Dirac cones are formed at all four high symmetry points, i.e., all $\bar{M}$ and $\bar{\Gamma}$ points. This is shown in Supplementary Fig. 11b. Upon Bi-doping, the valley degeneracy is lifted and a gap opens up at the $\bar{\Gamma}$ point, while the Dirac cones remain closed, i.e., ungapped at all three $\bar{M}$-points (Supplementary Fig. 11c). This makes Bi-doped $Pb_{1-x}Sn_xSe$ a Z_2 topological insulator with odd number of band inversions.

Supplementary Note 10: Core-level photoemission

Core-level spectra measured with 90 eV photons for $Pb_{1-x}Sn_xSe$ with 28% Sn concentration and 2.2% Bi doping are shown in Supplementary Fig. 12a. The overlap of Bi $5d_{5/2}$ with Sn $4d_{5/2}$ emission is resolved by a fitting procedure, the results of which are shown in Supplementary Fig. 12b. To further evaluate the stoichiometry from the core-level spectra, in particular the Bi concentration, we analyzed the intensity ratios by taking into account the peak areas and the corresponding photoemission cross sections of the individual elements [7]. Specifically, we used a similar fitting procedure as the one described in Supplementary Note 6. After normalizing the peak areas of individual elements by their photoemission cross section, we derived the Bi concentration from the ratio $n_{Bi}=A_{Bi}/(A_{Bi}+B_{Pb}+C_{Sn})$, where A, B, C denote the corresponding peak areas normalized with respect to the area under the Se peak. For the core-levels shown in Supplementary Fig. 12, we derive $A_{Bi}=0.0973$, $B_{Pb}=2.04129$ and $C_{Sn}=0.84593$, which yields an absolute value of $n_{Bi}\sim3.2$ % that is in fair agreement with the more accurate result obtained using high resolution x-ray diffraction and the Vegard's law (see Supplementary Fig. 2 and Supplementary Note 2).

Supplementary References

1. Springholz, G., Ueta, A. Y., Frank, N. & Bauer, G. Spiral growth and threading dislocations for molecular beam epitaxy of PbTe on BaF_2 (111) studied by scanning tunneling microscopy. *Appl. Phys. Lett*. **69**, 2822-2824 (1996).

2. McCann, P. J., Fuchs, J., Feit, Z. & Fonstad, C. G. Phase equilibria and liquid-phase-epitaxy growth of PbSnSeTe lattice-matched to PbSe. *J. Appl. Phys.* **62**, 2994-3000 (1987).

3. Ueta, A. Y., Springholz, G., Schinagl, F., Marschner, G. & Bauer, G. Doping studies for molecular beam epitaxy of PbTe and $Pb_{1-x}Eu_xTe$. *Thin Solid Films* **306**, 320-325 (1997).

4. Shirley, D. A. High-resolution x-ray photoemission spectrum of the valence bands of gold. *Phys. Rev. B* **5**, 4709 (1972).

5. Wojek, B. *et al*. Spin-polarized (001) surface states of the topological crystalline insulator $Pb_{0.73}Sn_{0.27}Se$. *Phys. Rev. B* **87**, 115106 (2013).

6. Xu, S.-Y. *et al*. Unconventional transformation of spin Dirac phase across a topological quantum phase transition. *Nature Commun.* **6**, 6870 (2015).

7. Yeh, J. J. & Lindau, I. Atomic subshell photoionization cross sections and asymmetry parameters: $1\leq Z \leq 103$, in *Atomic Data and Nuclear Data Tables*, **32**, 1-155 (1985).
